# Retinal Emboli in Cholesterol Crystal Embolism

**DOI:** 10.1155/2013/421352

**Published:** 2013-12-11

**Authors:** Antoine Rousseau, Ivan de Monchy, Emmanuel Barreau, Yasmina Yahiaoui, Mohamed M'garrech, Godefroy Kaswin, Marc Labetoulle

**Affiliations:** ^1^Department of Ophthalmology, Bicêtre University Hospital, Assistance Publique-Hôpitaux de Paris, South Paris University, 78 rue du Général Leclerc, 94275 Le Kremlin-Bicêtre Cedex, France; ^2^Department of Nephrology, Bicêtre University Hospital, Assistance Publique-Hôpitaux de Paris, South Paris University, 78 rue du Général Leclerc, 94275 Le Kremlin-Bicêtre, France

## Abstract

Cholesterol crystal embolism (CCE) is a rare and severe multisystemic disorder. It results from a massive release of cholesterol crystals from widespread atherosclerotic disease. The main difference with atherosclerosis is the severity and the quantity of the embolic events that occur during the course of the disease, eventually leading to multivisceral failure and death. The symptoms are multiple and make it a diagnostic challenge. Fundoscopic examination can be of great help, showing retinal emboli in up to 25% of the cases, and has been rarely described in the ophthalmologic literature. We report the case of a 77-year-old man with acute renal failure after coronarography. Retinal emboli seen in the fundus confirmed the diagnosis of cholesterol crystal embolism and thus prevented any further invasive investigations. In this case, anticoagulants must be stopped and any further endovascular procedure proscribed. Although impossible for this patient, peritoneal dialysis should be preferred to hemodialysis because it does not need any anticoagulation. Systemic corticosteroid can be used in the acute phase. Fundoscopic examination should be performed each time cholesterol crystal embolism is suspected. When typical emboli are seen in the retina, it permits avoiding invasive investigations and saving precious time for the management of this potentially lethal disease.

## 1. Introduction

Retinal arteriolar emboli may occur in about 1% of the general population and often reveal an underlying systemic vascular or cardiac disease [1, 2]. Hollenhorst first established the relationship between bright plaques in the retinal arterioles and atheromatous lesions of the aorta and the carotids [3]. More recently, large epidemiologic studies described the association of retinal arteriolar emboli with an increased risk of cerebrovascular morbidity and mortality. Based on the pathogeny and the fundoscopic appearance, retinal emboli are classified in several categories [4]. Calcic emboli are most often large, white and unique, and commonly impact into proximal retinal arterial branches. They originate from a calcified aortic valve or a calcified plaque of the carotid or the ascending aorta. Cholesterol emboli are small, yellowish, reflective, and often multiple. They localize at arteriolar bifurcations and may move and finally disappear following an ocular massage. They usually originate from an ulcerated atheromatous plaque within the carotid arteries and can cause a visual field defect when they block the vascular flow or remain asymptomatic. Other types of emboli (tumoral, fatty…) are less frequent [3, 5, 6]. Finally, retinal emboli can be associated with a rare entity, defined as cholesterol crystal embolism (CEE). In this disorder, emboli are multiple, involving several organs and leading to death in up to 73% of the cases [7].

Systematic review of the literature using PubMed database only found 3 publications relating cholesterol crystal embolism with ocular involvement [8–10]. The literature mainly focuses on carotid-related emboli, without differentiating this etiology from the other causes.

This is however a key point since this etiology of retinal cholesterol emboli implies a specific care. We report a case in which the fundoscopic examination was sufficient to confirm the diagnosis of cholesterol crystal embolism, avoiding the use of more injurious biopsies before the onset of specific management.

## 2. Case Presentation

A 77-year-old patient, with a high load of atherosclerotic risk factors (hypertension, smoker, hyperlipidemia, and diabetes mellitus) presented with an acute renal failure with conserved diuresis 3 weeks after undergoing coronarography to investigate a dyspnea on effort. The patient had undergone a quadruple coronary bypass and surgery of an abdominal aortic aneurysm, respectively, 13 and 18 years earlier. Serum creatinine before coronarography was 175 *μ*mol/L.

**Figure 1:**
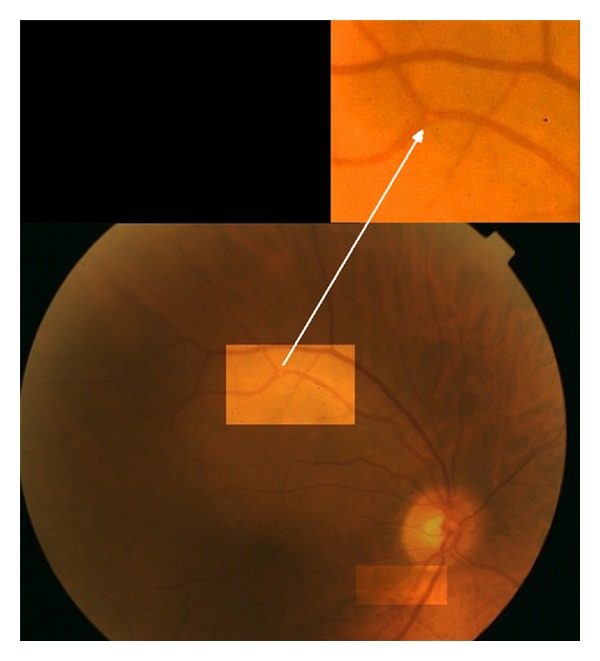
Right eye fundus photography of the patient: see the two emboli along the inferotemporal (highlighted area) and superotemporal (highlighted and magnified area) arcades.

At admission, there was neither *livedo reticularis* nor blue toe. Laboratory investigations revealed a serum creatinine level at 706 *μ*mol/L, with hyperkalemia (6 *μ*mol/L) and hypereosinophilia (1.070 × 10^9^ eosinophils/L). Two diagnoses were initially suspected: acute tubular necrosis following iodine-based contrast agent or renal atheroembolism. The patient was thus referred in the ophthalmology department to assess the latter hypothesis. Visual acuity was 20/25 for both eyes with no visual disturbance. Slit lamp examination found a mild and bilateral nuclear cataract. Fundus examination of the right eye revealed two arteriolar emboli along the superotemporal and inferotemporal arcades (Figure 1). The left eye fundus was normal. Renal failure was thus attributed to cholesterol crystal embolism. He was treated with periodic haemodialysis as peritoneal dialysis was impossible due to the presence of an eventration (secondary to his abdominal aortic aneurysm surgery). He died 5 months later because of mesenteric ischaemia induced by oral anticoagulation introduced 10 weeks earlier for atrial fibrillation.

## 3. Discussion

Cholesterol crystal embolism (CCE) is a severe disorder that usually affects several organs. It results from a massive release of cholesterol crystals from widespread ulcerative atherosclerotic disease of the aorta and/or its first branches [11, 12]. The main difference with atherosclerosis is the severity and the quantity of the embolic events that occur during the course. It mostly affects white Caucasians, more frequently men in their sixth decade, with cardiovascular risk factors and multiple manifestations of atherosclerosis [12, 13]. Embolization can occur spontaneously but an iatrogenic triggering event is noticed in up to 79% of cases (e.g., transluminal angioplasty, angiography, vascular surgery, and anticoagulant or fibrinolytic treatment) [12].

From a pathogenic point of view, CCE behaves like small vessels vasculitis. Crystals are too small to obstruct the artery in which they are located and induce an endothelial inflammatory reaction which leads to a complete obstruction within weeks or months [8, 12, 13]. The vasculitis may be associated with biologic features of inflammation and eosinophilia. Locations of emboli are multiple and thus cause proteiform clinical manifestations. The diagnosis is classically suspected in case of an acute renal failure associated with *livedo reticularis* and blue toe with preserved distal pulses, following any type of endovascular procedure [11]. Beside these typical settings, chronic forms of the disease may occur, with multiple embolizations, delayed from the triggering event. Masquerading presentations with essentially symptoms of vasculitides have been reported [8]. These multiple patterns make CEE a diagnostic challenge, and the diagnosis is often made only with autopsy [8, 9, 12]. The prognosis is poor, with death in up to 73% of the cases, secondary to multisystemic ischaemic complications, renal failure, sepsis, and cardiac arrest [7].

Ophthalmologic findings are the consequences of retinal emboli (monocular transient or definitive vision loss and visual field defects) or may be related to secondary vasculitis (diplopia caused by neurologic or muscular lesions and central neuro-ophthalmologic symptoms caused by visual paths lesions) [8, 9]. The ocular complications of CEE have seldom been described in the ophthalmologic literature, despite the putative role of fundus examination to confirm the diagnosis. Prevalence of retinal emboli varied from 10% to 25% [10]. Emboli can be multiple and disseminated or unilateral [8–10]. This heterogeneity can be explained by the locations of the plaques (retinal emboli occurs only if the plaques are located either on the carotid arteries or on the aorta), but also by the mobile nature of these emboli. Fundus examination has a double interest in suspected cases of CEE. In a patient with visceral failure, it confirms the hypothesis and makes useless invasive biopsies, as it was the case in our patient and in those reported by Gittinger and Kershaw [10]. Systematic fundus examination also enables exploring the CEE hypothesis in case of vasculitis or unexplained neuro-ophthalmologic signs, which could be initially misdiagnosed as giant cell arteritis, for example, as reported by Jacobson [8].

Since retinal emboli of CEE appear as usual cholesterol emboli detached from carotid atheromatous plaques, it is of crucial importance to ask the patient for a previous history of vascular procedure. The main differences lie both in the short-term prognosis and the management: CEE can quickly lead to multivisceral failure, whereas usual retinal emboli are only associated with a higher risk of stroke and delayed mortality [1, 2, 14]. Once the diagnosis of CEE is established, any form of anticoagulation or endovascular procedure should be banned. Some authors have used steroids and/or statins [13, 15]. Although a particular dialysis option is not contraindicated, some investigators suggest that peritoneal dialysis, which avoids anticoagulation, could be the preferred treatment modality [12]. Prospective studies reported an improvement of the survival rate with supportive and adapted treatments [15].

Fundoscopic examination may be the key test to diagnose cholesterol crystal embolism. When typical emboli are seen in the retina, it obviates the need for invasive investigations, thus saving precious time to manage this potentially lethal disease.
